# Supplementary material for: Investigating associations between COVID-19 mortality and population-level health and socioeconomic indicators in the United States: A modeling study
Source: PLoS Med. 2021 Jul 13;18(7):e1003693. doi: 10.1371/journal.pmed.1003693 (PMC8277036; doi:10.1371/journal.pmed.1003693)
Supplement: S1 Text — Fig A: Distribution of crude COVID-19 mortality rates (deaths per thousand residents) for each of 8 age groups in US states. Each data point indicates a US state, and the bounded region indicates the distribution. Note that the y-axis is on log10 scale. Fig B: Scatter plot of crude (y-axis) and age-standardized (x-axis) mortality rates in US states. A state below the diagonal (black dashed line) indicates that the mortality rate increases when standardized. Fig C: Effect estimates (95% CI) with a linear univariate linear model using crude mortality rate (red) and age-stratified mortality rate (in green) as response variables. Labels indicate adjusted R2. Inset magnifies select variables of smaller estimates. All estimates are significant (p < 0.05). The slight difference between the effect estimates with crude rates here and Fig 5 of the main text is due to the use of different data sources (The New York Times in the main text and NCHS provisional counts here). Fig D: Variables estimates of spatial lag models built using crude (orange) and age-standardized (brown) rates as the response variable. p-values indicated when p > 0.05. adj.rsq: adjusted R2; rho: spatial lag coefficients. COVID, Coronavirus Disease 2019. (DOCX) [file pmed.1003693.s002.docx]

**Investigating associations between COVID-19 mortality and population level health and socioeconomic indicators in the United States: A modeling study**

***Supplementary Text***

Sasikiran Kandula, Jeffrey Shaman

Department of Environmental Health Sciences, Columbia University, New York, New York.

*Age-standardized mortality rates*

The case and mortality rates used in the main text of this study are crude rates that did not account for differences in age distribution across counties. Adjusting for age distribution has not been possible because public sources of age-stratified count data at the county level have been unavailable. Here we describe a method to approximate age-standardization using state-level mortality data and report results from univariate and spatial models with these rates as the response variable.

Let $p_{s}^{a}$ denote the population in age group *a* and state *s* and $d_{s}^{a}$ the corresponding COVID-19 deaths through December 31, 2020. The population estimates were obtained from US Census Bureau’s 2019 postcensal estimates (1) and mortality data are provisional estimates from the National Center for Health Statistics (NCHS) (2). Fig A shows distribution of mortality rates, ${d_{s}^{a}}/{p_{s}^{a}}$, in US states for 8 age groups – under 25 years, 10-year groups for ages between 25-85 years and 85+ years. The overall unadjusted mortality rate for a state is given by $\frac{\sum_{a} d_{s}^{a}}{\sum_{a} p_{s}^{a}}$

To adjust for differences in age distribution, we standardize to US national level age distributions. A state’s age-standardized mortality rate is calculated as $\sum_{a} \left( \frac{d_{s}^{a}}{p_{s}^{a}}*\frac{\sum_{s} p_{s}^{a}}{\sum_{a,s} p_{s}^{a}} \right)$. Fig B shows a comparison of state level crude and age-standardized rates.

At the county level, let $p_{c}^{a}$ and $d_{c}^{a}$ be the population and deaths in county c. $d_{c}^{a}$ is unavailable, but $d_{c}$, total deaths across all age groups, and $p_{c}^{a}$ are known from which we estimate $d_{c}^{a}= d_{c}*{d_{s}^{a}}/{\sum_{a} d_{s}^{a}}$ where *c* ∊ *s*, i.e. we assume that the proportion of deaths occurring in an age group is the same for all counties in the state and is identical to the state’s proportion. This allows estimation of counties’ age-standardized rates analogous to states’ as $\sum_{a} \left( \frac{d_{c}^{a}}{p_{c}^{a}}*\frac{\sum_{s} p_{s}^{a}}{\sum_{a,s} p_{s}^{a}} \right)$ .

Fig C shows a comparison of effect estimates of the indicators with crude (similar to Fig 5 of the main text) and age-stratified mortality rates. Key observations:

- The proportion of the variability explained by an indicator (Adjusted R^2^) is higher with age-stratified rates than with crude rates for nearly all indicators (with the exception of *nursing home residents*)
- The effect estimates for indicators are also higher with age-stratified rates except for the two indicators that are more directly related to age, proportion of *nursing home residents* and proportion *elderly.* While with the former the change is a decrease in the magnitude of association, for the *elderly* indicator, the direction of association was found to have reversed with the standardization of mortality rates.

Fig D shows estimates from two spatial lag models built using crude and age-standardized rates as response. Consistent with the univariate analysis, the association of the *elderly* indicator has been reversed with standardization and that of *nursing home residents* decreased considerably. There are no large changes in other indicators. The lag coefficient is nearly similar for the two models and adjusted R^2^ is higher for the age-stratified model.

Together these results suggest that the findings presented in the main text may not be sensitive to differences in age distributions in counties, but this remains to be verified when age-stratified county mortality counts become available.

**References**

1. National Center for Health Statistics. Vintage 2019 postcensal estimates of the resident population of the United States for April 1, 2010, July 1, 2010-July 1, 2019. Prepared under a collaborative arrangement with the U.S. Census Bureau. Available from: http://www.cdc.gov/nchs/nvss/bridged_race.htm

2. National Center for Health Statistics. Provisional Death Counts for Coronavirus Disease 2019 (COVID-19): Weekly Updates by Select Demographic and Geographic Characteristics.

<https://www.cdc.gov/nchs/nvss/vsrr/covid_weekly/index.htm>


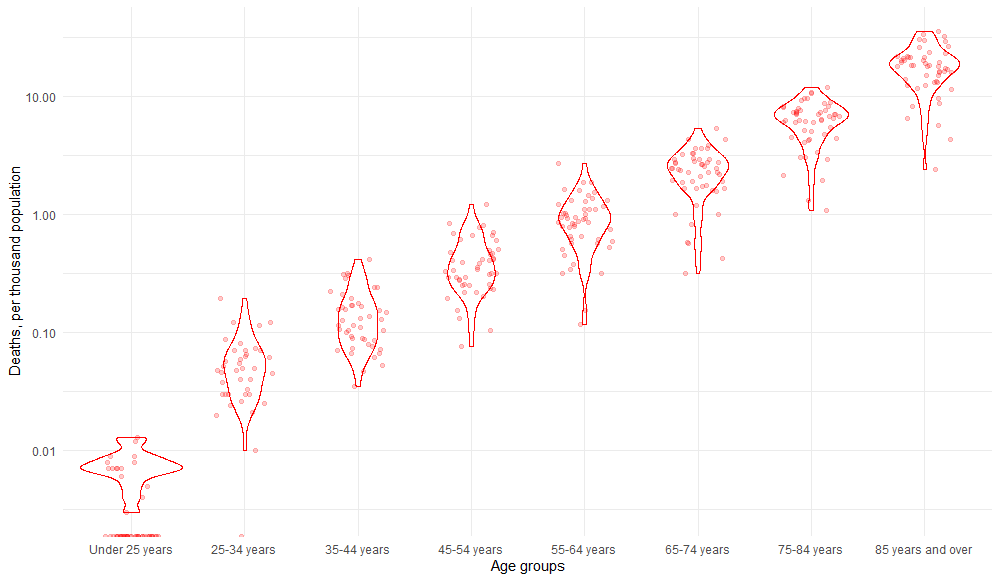


**Fig A.** Distribution of crude COVID-19 mortality rates (deaths per thousand residents) for each of 8 age groups in US states. Each data point indicates a US state and the bounded region indicates the distribution. Note that the *y*-axis is on log_10_ scale.


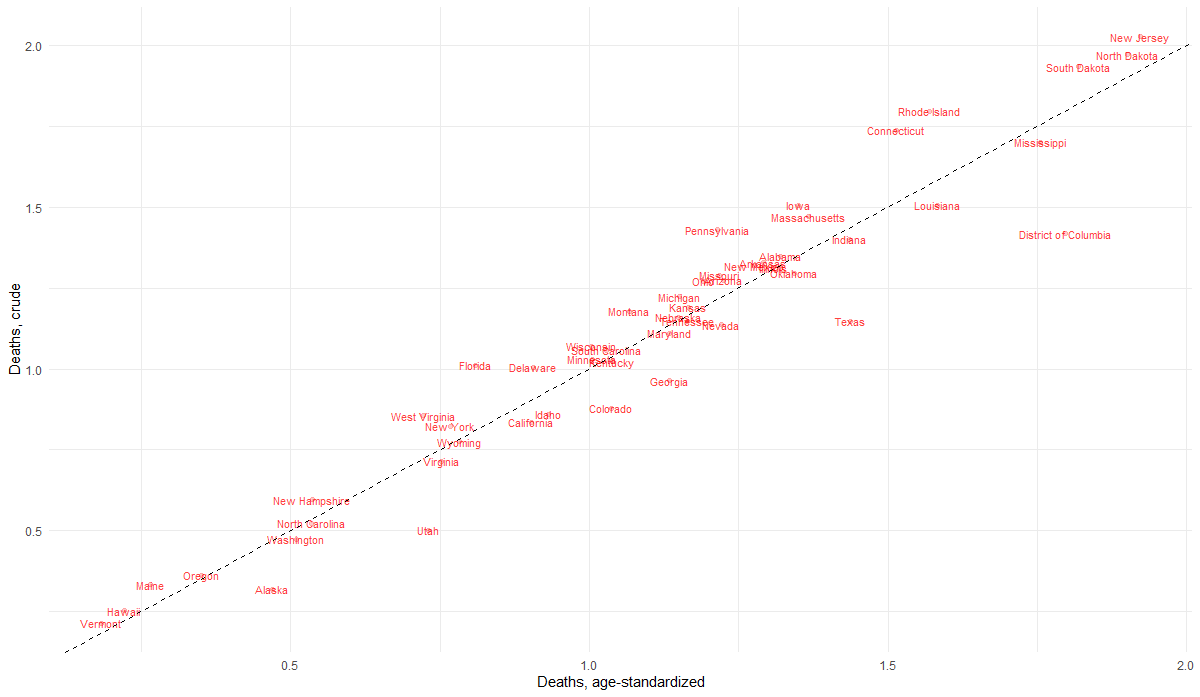


**Fig B**. Scatter plot of crude (y-axis) and age-standardized (x-axis) mortality rates in US states. A state below the diagonal (black dashed line) indicates that the mortality rate increases when standardized.


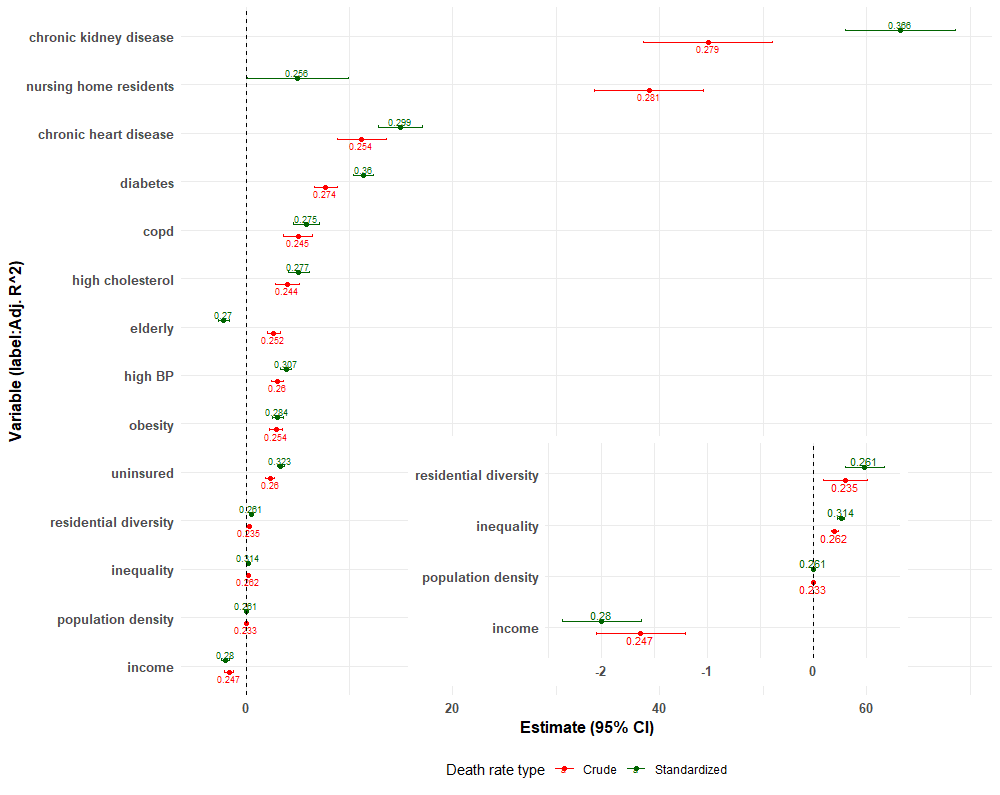


**Fig C.** Effect estimates (95% CI) with a linear univariate linear model using crude mortality rate (red) and age-stratified mortality rate (in green) as response variables. Labels indicate adjusted R^2^. Inset magnifies select variables of smaller estimates. All estimates are significant (*p* < .05). The slight difference between the effect estimates with crude rates here and Figure 5 of the main text is due to the use of different data sources (New York Times in the main text and NCHS provisional counts here)


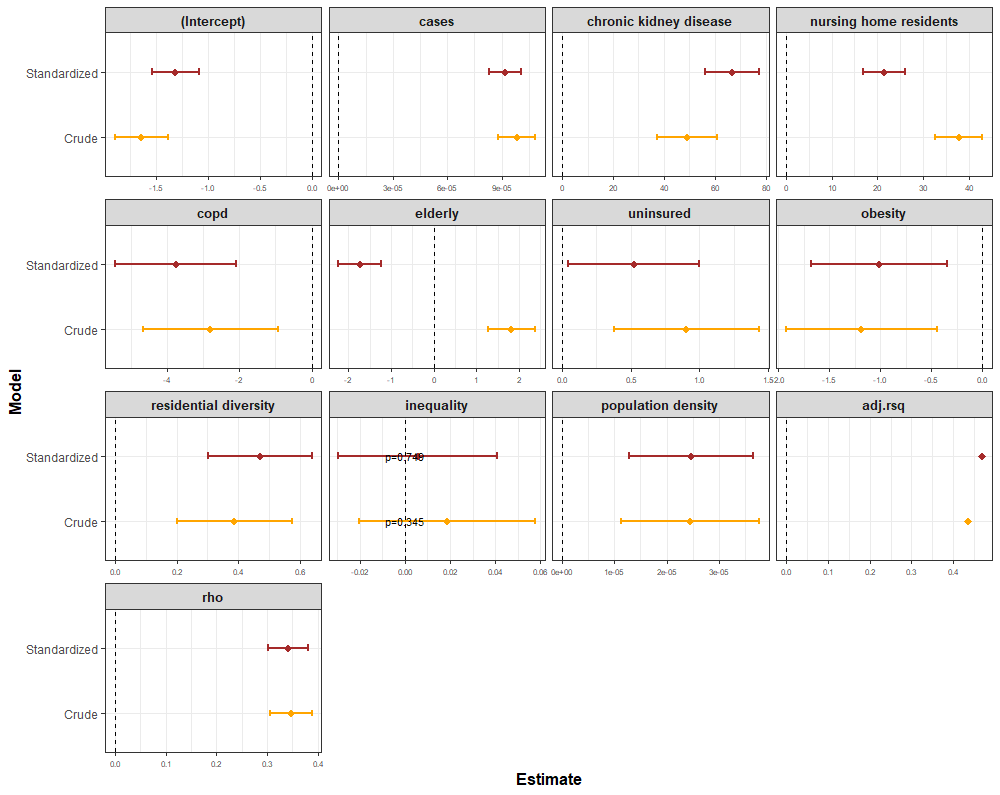


**Fig D**. Variables estimates of spatial lag models built using crude (orange) and age-standardized (brown) rates as the response variable. *p*-values indicated when *p* > .05. *adj.rsq*: Adjusted R^2^; *rho*: spatial lag coefficients
